# Systemic Transcriptional Responses to Clinical Vaccine Formulations and Live-Attenuated Vaccination: Comparative Kinetics and Hypotheses for Therapeutic Cancer Vaccine Monitoring

**DOI:** 10.3390/vaccines14090792

**Published:** 2026-09-09

**Authors:** Corey K. Goldman

**Affiliations:** 1 Department of Cardiovascular Medicine, MedAlliance, Bronx, NY 10458, USA; coreykeith31@gmail.com; 2 Leon H. Charney Division of Cardiology, Department of Medicine, NYU Grossman School of Medicine, NYU Langone Health, New York, NY 10016, USA

**Keywords:** vaccine adjuvant, AS01, AS03, MF59, yellow fever vaccine, systems vaccinology, transcriptomics, cancer vaccine, antigen presentation, immune monitoring

## Abstract

Background/Objectives: Therapeutic cancer vaccination requires innate sensing, antigen presentation, lymphocyte priming, tumor access, cytotoxicity, and persistence. Prophylactic studies cannot demonstrate these antitumor functions but can compare systemic formulation responses. We tested whether blood transcription extended beyond innate responses to cancer-immunity-cycle programs. Methods: From 385 GEO records, we retained six human blood cohorts (five bulk-RNA and one sorted-cell) and one mouse blood/lymph-node study. We percentile-ranked 23 modules and compared baseline changes across 17 formulation questions. A separate screen of 35 poly-ICLC cancer-vaccine studies yielded NCT01204684; all arms received a tumor-lysate-pulsed dendritic-cell vaccine. Results: At 24 h after dose 2, AS01B, AS01E, and AS03 increased type I interferon-associated transcription relative to aluminum salt; the AS04 change was negligible. MF59 increased this score by 3.2 points (95% confidence interval, 1.2–5.1) relative to unadjuvanted antigen. Responsive comparisons also showed higher transcription of genes associated with antigen-presenting-cell costimulation, type 1 conventional dendritic cells, and MHC-I antigen processing/presentation. Yellow fever 17D produced a multicomponent trajectory, but the comparison did not isolate viral replication, antigen persistence, viral sensing, or tissue distribution. Of 390 evaluable module–formulation combinations, 278 had no result after false discovery rate (FDR) correction. Sensitivity estimates agreed (r = 0.998; 97.8% directional agreement). In NCT01204684, poly-ICLC recipients showed within-arm interferon and antigen-processing changes, but none of 46 agonist-versus-placebo module contrasts survived FDR correction. Conclusions: Early systemic transcription differed among formulations, whereas later cancer-immunity programs were infrequently detected in bulk blood RNA. Therapeutic studies should add draining-node or tumor measurements and antigen-specific functional assays.

## 1. Introduction

Therapeutic cancer vaccination succeeds only if several biologically distinct events occur in sequence. Innate sensing and antigen uptake must be followed by antigen processing, costimulation, antigen-specific lymphocyte priming and expansion, trafficking into tumor, recognition of naturally processed antigen, cytotoxic function within a suppressive microenvironment, and persistence [1,2,3,4]. An early cytokine or transcriptional response can document initiation of this sequence, but it cannot establish completion.

Clinical prophylactic-vaccine trials provide a useful but limited reference for the initiation of this sequence. When participants in treatment and reference arms are sampled in parallel and receive the same antigen, the between-arm contrast estimates the systemic increment associated with adding or changing an adjuvant. Vaccine-versus-placebo contrasts instead measure the combined response to antigen plus formulation. Both designs contain fewer disease- and treatment-related confounders than cancer cohorts, but neither makes healthy participants surrogates for patients with cancer or supplies evidence of tumor control [5,6,7,8].

Interpreting those blood measurements requires separating a transcriptional association from a biological function. Whole-blood modules combine changes in gene expression with changes in leukocyte composition, and genes associated with dendritic cells, germinal centers, cytotoxic lymphocytes, or stromal programs are not cell-specific functional assays. Conversely, failure to detect a later program in blood is a finding about the sampled compartment and schedule, not proof that the program was absent from a draining lymph node or tumor. These limits are particularly important when using systemic data to formulate hypotheses for therapeutic vaccination.

Live-attenuated yellow fever vaccine 17D provides a useful kinetic reference because its response has been characterized extensively and one included trial measured it on the same platform and schedule as several other vaccines [9,10,11]. Yellow fever 17D differs from nonreplicating formulations in viral replication, antigen persistence, the number and timing of pattern-recognition inputs, and anatomical distribution. Its longer systemic response occurred together with viral replication, antigen persistence, multiple innate inputs, and broader tissue distribution; this study did not isolate any one of these features.

This comparative reanalysis addresses four questions. First, which clinical formulations produce reproducible early systemic transcriptional responses when matched controls are available? Second, how do the kinetics of those responses compare descriptively with yellow fever 17D? Third, across 23 modules selected to represent transcriptional programs relevant to distinct requirements of therapeutic vaccination, which programs are detected in blood and which are not? Fourth, which unmeasured or compartment-limited steps should be tested directly in therapeutic cancer-vaccine trials? The study does not test cancer-vaccine efficacy; it uses controlled prophylactic-vaccine data to define what early systemic blood measurements establish and to formulate prospective hypotheses. A separate public-data census and harmonized reanalysis place poly-ICLC in a therapeutic cancer-vaccine context without pooling that small, clinically heterogeneous study with the healthy cohorts.

## 2. Materials and Methods

### 2.1. Dataset Identification, Eligibility, and Analytic Scope

**Figure 1:**
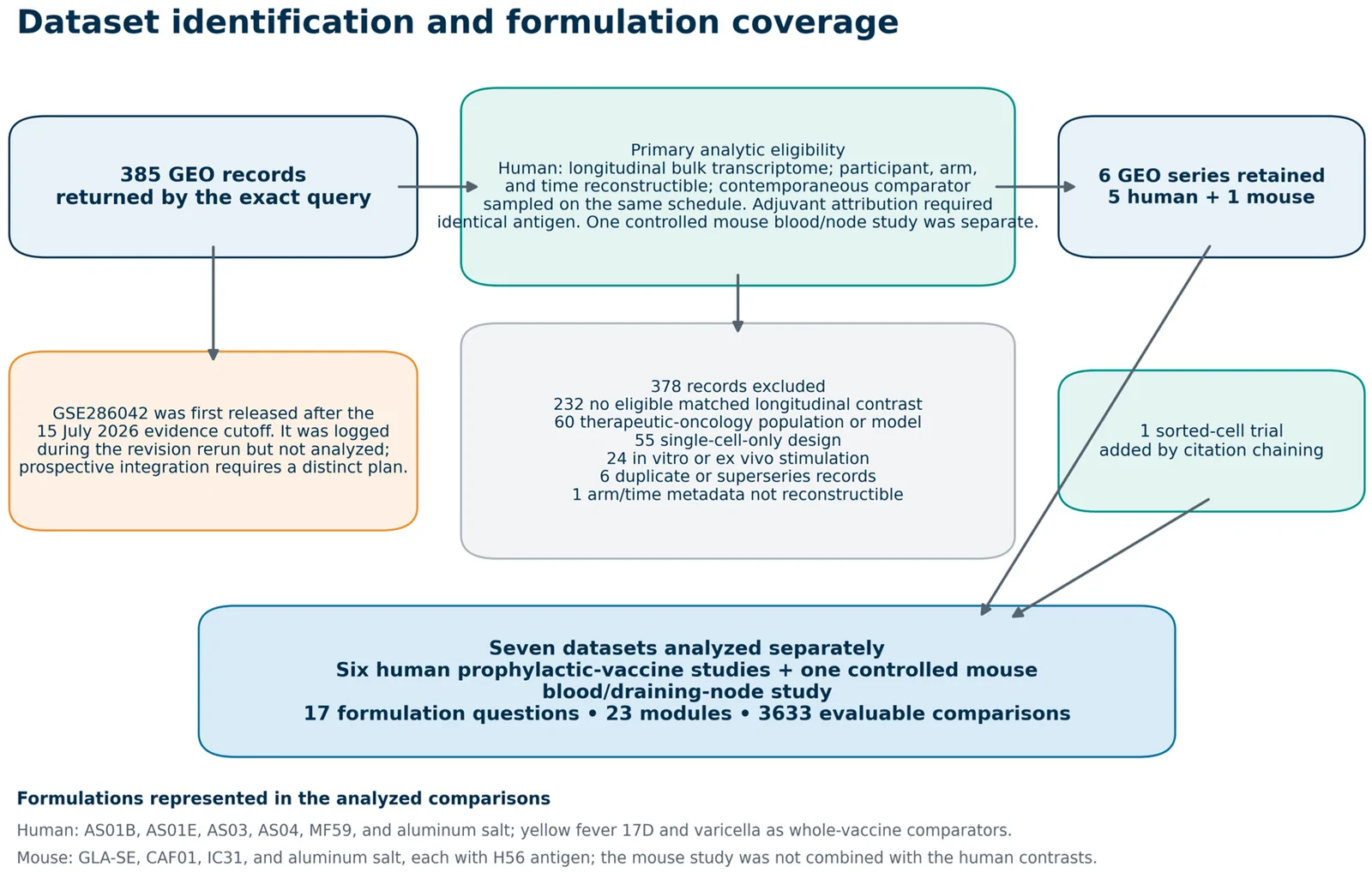
**Dataset identification and formulation coverage.** The evidence cutoff was 15 July 2026, and the exact GEO query was rerun on 23 August 2026 to reproduce the record census. Five human GEO series and one controlled mouse GEO series were retained, and one human sorted-cell trial was added by citation chaining. The resulting evidence base, therefore, comprises six human prophylactic-vaccine studies and one controlled mouse compartment study. GSE286042 was first released after the cutoff and was logged but not analyzed; prospective integration requires a distinct normalization and hierarchical analysis plan. The lower panel names every adjuvant or formulation represented in the retained comparisons. The accession-level ledger and exact query are provided in Table S12.

Public records were sought in the NCBI Gene Expression Omnibus (GEO) for human or mouse expression-profiling studies involving vaccination, immunization, or named clinical adjuvants. The primary estimand was restricted to controlled prophylactic vaccination in healthy participants; therapeutic-oncology populations and models were catalogued during screening but were not pooled into that primary evidence base. The evidence cutoff was 15 July 2026. On 23 August 2026, the query was rerun through the NCBI GDS E-utilities interface to reproduce the record census; records first released after the cutoff were logged but not analyzed. The exact query is reported in Table S12 and returned 385 records. Human records were eligible if they provided longitudinal bulk transcriptomic measurements; reconstructible participant, treatment arm, and sampling-time metadata; and a contemporaneous comparator arm sampled on the same schedule. Adjuvant-attributable comparisons additionally required the same antigen in both arms. Vaccine-versus-placebo comparisons were retained but interpreted as the combined response to antigen plus formulation. One controlled mouse study was retained separately because it sampled both blood and draining lymph node after the same immunization; it was not combined with the human comparisons. Figure 1 reports the selection counts and all formulations represented in the retained studies, and Table S12 provides the accession-level decision ledger.

Five human GEO series and one controlled mouse GEO series met these criteria. A randomized human cell-sorted trial was added by citation chaining, yielding six human prophylactic-vaccine datasets and one mouse dataset. Of the 385 records returned by the revision query, 378 failed the stated design or metadata criteria and six were retained GEO series. GSE286042 was not included in the submitted analysis and was first released after the 15 July 2026 evidence cutoff; it was identified during the revision rerun and recorded for prospective validation because integration requires a distinct normalization and hierarchical analysis plan. The selection was designed to identify estimable formulation contrasts; it was not a systematic review or meta-analysis of vaccine efficacy.

### 2.2. Included Datasets

Each study was analyzed separately; no expression matrices or participant-level observations were pooled across studies. The six human datasets comprise healthy participants receiving influenza, hepatitis B, yellow fever, or varicella vaccines. The mouse study used subcutaneous tail-base immunization and terminal sampling; it is reported separately and is not treated as corroboration of a human effect. The included datasets are summarized in Table 1.

For GSE112293, 898 profiles had module scores. Five profiles lacked a compartment label sufficient to assign them to the separate whole-blood or PBMC analyses; the primary compartment-stratified comparisons, therefore, used 893 profiles. The five sample identifiers and disposition are reported in Table S1.

### 2.3. Module Selection, Terminology, and Scoring

Twenty-three modules were selected to evaluate transcriptional programs associated with distinct requirements across the cancer-immunity cycle, rather than to sample immune activation generically. The panel includes innate sensing and inflammation; APC readiness and peptide-presentation machinery; and lymphocyte priming, differentiation, trafficking, and adhesion. Additional modules represent innate training, tissue repair, and myeloid, stromal, metabolic, and vascular barriers relevant to tumor access and function. Table S2 lists every gene and source. Representative genes make the questions concrete: BATF3, CLEC9A, and XCR1 mark type 1 conventional dendritic cell (cDC1)-associated biology; CD40, CD80, and CD86 represent APC costimulation; HLA-A, B2M, TAP1, and PSMB8 represent MHC-I antigen-processing and presentation machinery; PRF1 and GZMB represent cytolytic machinery; CXCR3, CXCR6, ITGA4, and ICAM1 represent trafficking; and ARG1, TREM2, FAP, TGFB1, VEGFA, and ANGPT2 represent suppressive or exclusionary tumor-associated states. These are transcriptional associations, not cell-specific functional assays. The former “dendritic-cell licensing” score is, therefore, reported as APC-costimulation/cDC1-associated transcription, and the former “germinal-center response” is reported as GC/plasmablast-associated transcription.

**Figure 2:**
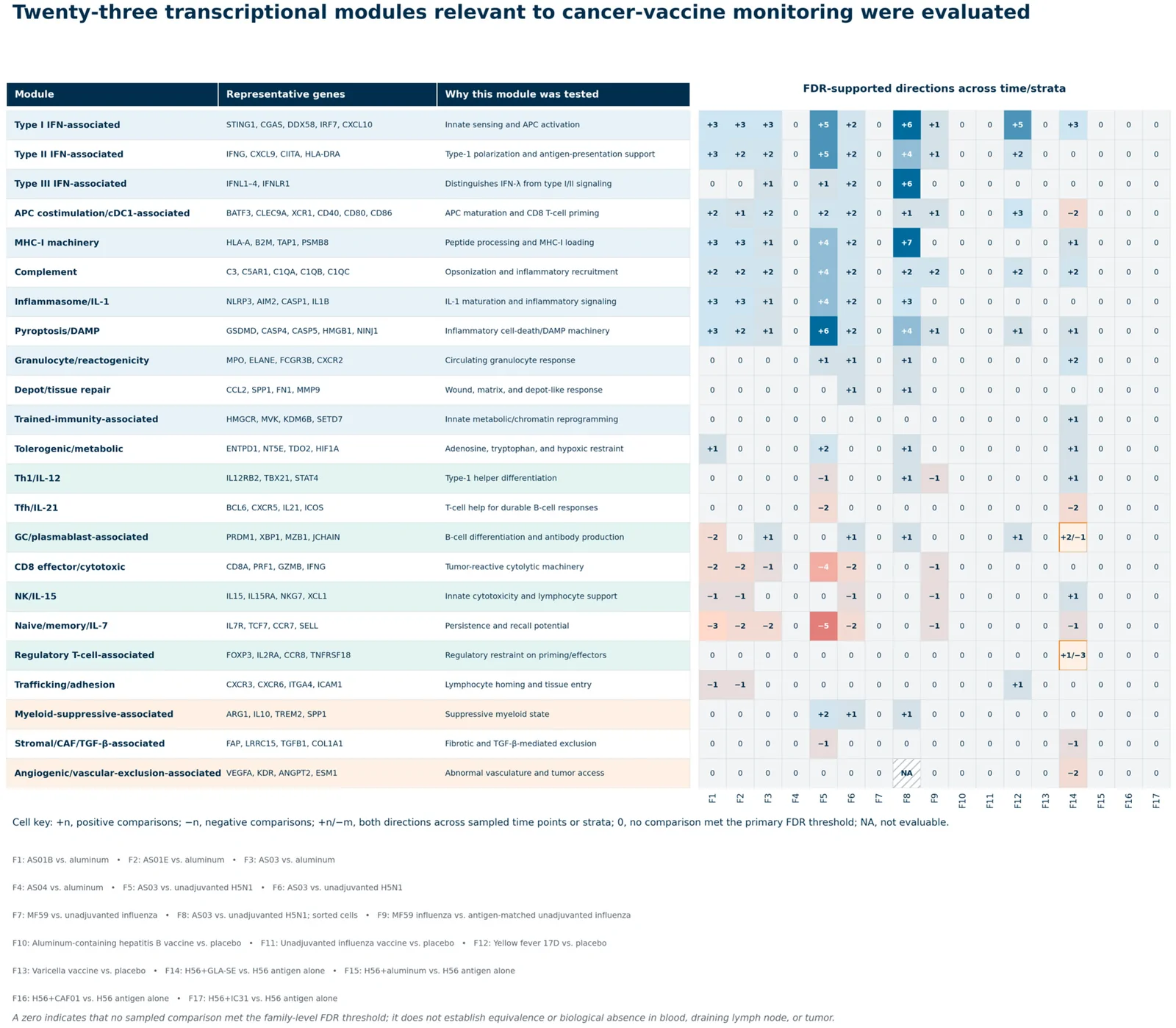
**Complete 23-module evidence matrix across 17 formulation questions.** The left panel names every module, representative genes, and the reason each associated transcriptional program was selected for cancer-vaccine monitoring. The right panel summarizes all sampled time points and strata after primary family-level FDR control. Each cell reports the number and direction of comparisons meeting that threshold: +n, positive; −n, negative; +n/−m, both directions across sampled time points or strata; 0, none; NA, not evaluable. The 278 explicit zeros among 390 evaluable cells show that the analysis evaluated transcriptional programs spanning the cancer-immunity cycle and did not report only responsive immune modules. A zero means that no sampled comparison met the threshold; it does not establish equivalence or biological absence. Blue cells indicate positive FDR-supported comparisons, coral cells indicate negative comparisons, pale-gray cells indicate no FDR-supported comparison, orange-outlined cells contain both directions, and hatched cells are not evaluable.

Figure 2 presents the rationale for each module alongside the complete module-by-formulation result matrix. Each of the 391 cells represents one module and one of the 17 formulation questions. A signed integer reports how many sampled time/stratum comparisons met the primary family-level FDR criterion in the positive or negative direction; an explicit zero reports that none did. Of 390 evaluable cells, 278 contained zeros and 112 contained at least one FDR-supported result; one sorted-cell module was not evaluable. The zeros document that the complete panel was evaluated, including many module–formulation cells without an FDR-supported change. No signal in blood at sampled times does not establish absence from draining lymph node or tumor.

Within each sample, expression features were converted to percentile ranks with average ranks for ties. Probes were mapped to gene symbols using the official platform annotation; ranks from multiple probes mapping to the same gene were averaged, and each module score was the mean rank of its observed genes. A module required at least three mapped genes and at least 40% of its defined membership. This single-sample rank strategy reduces sensitivity to platform scale and monotonic transformations and is related to established rank-based single-sample scoring methods [16,17,18]. It does not model gene-gene covariance, and its inferential unit is the participant-level score rather than the individual gene.

The 23 modules contain 298 memberships representing 290 unique genes. Eight genes occur in two modules, 6 of 253 module pairs overlap, and the maximum pairwise Jaccard index is 0.071 (Table S8). Across the four human array platforms, coverage ranged from 40% to 100%; every module met both scoring thresholds (Table S9).

### 2.4. Contrasts, Repeated Measures, Effect Sizes, and Multiplicity

For each human participant, module, and post-dose time point, the primary analysis calculated change from that participant’s sample immediately before the relevant dose. The formulation-associated effect is the difference in the mean participant-level change between arms. Welch’s t test was used because arm sizes and variances differed. Antigen-matched unadjuvanted vaccine was the reference where available; protocol-matched placebo was otherwise used. Within GSE124533, MF59-adjuvanted influenza was compared with antigen-matched unadjuvanted influenza, whereas the other vaccine arms were compared with the pooled placebo group defined by the BIOVACSAFE program. Antigen-matched contrasts estimate the incremental response to adding or changing an adjuvant because the antigen is identical. Vaccine-versus-placebo contrasts estimate the combined response to antigen plus formulation. Neither identifies a specific pattern-recognition receptor or distinguishes direct innate sensing from formulation-associated local tissue effects.

For each contrast, the revision reports the mean difference, 95% confidence interval, and Hedges g with its small-sample correction. Benjamini–Hochberg control at 0.05 was applied within each immunogen-versus-control family across all modules, time points, and sampled strata [19]. Each family corresponds to one formulation question within one study; the seven studies yielded 17 families. This primary rule identified 234 significant results among 3633 evaluable comparisons. Because grouping every comparison from the same study is more conservative and tests a broader question, a sensitivity analysis used one multiplicity family per study. It identified 210 results, with 28 rows changing classification. Both adjusted probabilities are retained in Table S5.

Because four human datasets contained dense repeated sampling, a sensitivity analysis fit Gaussian generalized estimating equations with an exchangeable working correlation and participant as the clustering unit [20]. The model included arm, categorical time, and arm-by-time terms; the interaction estimates the same baseline-referenced between-arm contrast while accounting explicitly for within-participant correlation. False-discovery control used the same family definitions as the primary analysis. GSE74975 used staggered sampling, and the cell-sorted trial added a second within-participant population level; their primary participant-level contrasts were retained rather than forcing a mismatched longitudinal model.

Robustness to the score definition was tested in GSE116975 by averaging probe expression to genes, standardizing each gene across the study, and taking the mean z-score of observed module genes. The same baseline-referenced contrasts and multiplicity families were then recomputed. This analysis was chosen as a transparent conventional alternative rather than as a claim that one scoring method is universally preferable.

### 2.5. Cellular-Composition Analyses

Lineage scores were constructed from cell-identity transcripts that were not selected as interferon-response genes (Table S3). Partial associations were calculated by regressing both the module of interest and the interferon-associated score on the relevant lineage index and correlating the residuals. A separate analysis regressed the interferon-associated score on monocyte and neutrophil lineage indices and repeated the arm comparison on residuals. These analyses assess how the associations change after accounting for estimated lineage scores; they neither quantify cell abundance nor establish a causal contribution. The sorted-cell study provides the stronger lineage-intrinsic test because changes within a purified population cannot be produced by shifts in the proportions of major blood-cell classes.

### 2.6. Serum Proteins

Public ImmPort data from SDY1252 provided 76 serum analytes in 39 participants at 14 time points after AS03-adjuvanted or unadjuvanted H5N1 vaccination. Dose-specific baseline changes were compared between arms with Welch tests and false-discovery control across the analyte panel. Leave-one-participant-out analyses and re-estimation from observed concentrations assessed sensitivity.

### 2.7. Poly(I:C)/Poly-ICLC Public-Transcriptome Availability Census

A separate descriptive census of GEO transcriptomic records involving poly(I:C), poly-ICLC, and related double-stranded-RNA agonists was frozen on 18 July 2026. The named search terms were poly(I:C), poly I:C, polyIC, poly-ICLC, Hiltonol, BO-112, rintatolimod, and TLR3 agonist. Official GEO Series, Sample, and Platform records were screened, with linked primary publications used to resolve formulation, route, timing, and study identity. Each unique GEO accession was the screening unit. An accession could contribute more than one accession–compartment coordinate when it contained distinct tissues or assays, but linked subseries, superseries, and accessions from the same trial/publication were collapsed under one duplicate-study key for study-level counts.

An in vivo study administered the dsRNA agonist to an intact human or nonhuman organism. An adaptive-phase study included at least one sampled time point seven or more days after administration or a paired vaccine-cycle comparison spanning such a time point. A vaccine/immunotherapy context used antigen-directed vaccination or cancer immunotherapy rather than direct cell stimulation, maternal immune activation, or another inflammatory model. A human blood study required peripheral blood or PBMC transcriptomics with a reconstructible contemporaneous treatment comparison. The census yielded 38 eligible accession–compartment coordinates from 35 unique GEO accessions, representing 32 independent studies.

The one human blood study meeting this controlled-comparison definition, NCT01204684 (GSE237562), was then reanalyzed as a separate cancer-specific extension. All randomized participants received ATL-DC; the randomized add-on treatment was placebo, poly-ICLC, or resiquimod. The public DESeq2-normalized, log2-transformed bulk-PBMC matrix was restricted to the 5, 8, and 8 baseline/post-treatment pairs that passed the published quality-control procedure. S27-06-2 (poly-ICLC) and S04-10-3 (resiquimod) were excluded because their post-treatment libraries had low complexity, as specified in Supplementary Data S1C of the source publication [21]. The mixed-treatment participant was not included.

The previously defined 23 modules were scored with the same within-sample percentile-rank procedure and coverage thresholds used in the primary analysis. For each participant, the baseline score was subtracted from the post-treatment score. The randomized estimands were the incremental effects of adding poly-ICLC or resiquimod to ATL-DC, each compared with ATL-DC plus placebo. Each agonist-versus-placebo comparison was treated as a separate 23-test multiplicity family. Effects are reported in percentile points with 95% Welch confidence intervals, Hedges g, and Benjamini–Hochberg-adjusted probabilities. Exact label-permutation tests and the alternative mean-z module score were sensitivity analyses. Because two of the five placebo post-treatment samples were obtained on day 15, whereas the active-arm samples were predominantly day 29 (one resiquimod sample was day 35), a further sensitivity analysis restricted the placebo reference to the three day-27/29 pairs. The direct poly-ICLC-versus-resiquimod contrast and within-arm baseline/post-treatment changes were secondary; neither isolates the effect of ATL-DC itself. No tumor, response, progression-free-survival, or overall-survival outcome was reanalyzed.

### 2.8. Software, Data, and Use of Generative AI

The primary submitted analysis used Python 3.10.12 with numpy 2.2.6, pandas 2.3.3, scipy 1.15.3, statsmodels 0.14.6, and matplotlib 3.10.9. The separate NCT01204684 extension used Python 3.12.13 with numpy 2.3.5, pandas 2.2.3, and scipy 1.17.0. Revision analyses used deposited per-sample scores, public normalized expression matrices, and official repository annotations. The submitted analysis, derived scores, code, supporting documentation, and final Supplementary Materials workbook are archived at https://zenodo.org/records/22133852 (accessed on 7 July 2026).

During the manuscript’s preparation and revision, the author used OpenAI ChatGPT 5.6 and GPT-5.6-Codex (OpenAI, San Francisco, CA, USA; web-based service versions current during July–August 2026) to assist with code implementation and debugging under author-defined analysis plans, organization of public data and references, figure implementation, and language editing. AI-generated code, prose, and summaries were treated as unverified drafts. The author defined the biological questions, modules, contrasts, and interpretation; inspected and verified the code and analytic outputs against the source data; evaluated the literature; selected the final analyses; and accepts full responsibility for the manuscript. This task-specific disclosure follows the journal’s requirement to state where and how generative AI was used (ChatGPT 5.6).

## 3. Results

### 3.1. Study Selection and Analytic Checks Yielded a Deliberately Narrow Evidence Base

The 23 August 2026 GEO rerun returned 385 records against an evidence cutoff of 15 July 2026. Five human GEO series and one controlled mouse GEO series satisfied the stated criteria, one human cell-sorted trial was added by citation chaining, and 378 records failed the design or metadata criteria. GSE286042 was first released after the cutoff, was logged but not analyzed, and requires a distinct normalization and hierarchical analysis plan for prospective integration. The resulting primary evidence base is deliberately narrow: it contains randomized or controlled formulation comparisons capable of estimating systemic responses under healthy prophylactic-vaccine conditions. A cancer-specific poly-ICLC trial is considered separately in Section 3.8 because its participants, intervention, and sampling schedule answer a different question.

Module overlap was low, and every human platform-by-module combination met the prespecified coverage threshold; neither finding explains the observed response patterns. Only 8 of 290 genes occurred in more than one module, and no pair shared more than 7.1% of its combined membership. All 92 human platform-by-module cells met the coverage floor, with a range of 40–100%. The lower-coverage cells were retained under the stated scoring rule rather than selectively excluded.

The complete result matrix also shows the breadth of testing beyond early immune-response markers. Across 23 modules and 17 formulation questions, 278 of 390 evaluable module-by-family cells contained no FDR-supported result at any sampled time or stratum, 112 contained at least one, and one cell was not evaluable (Figure 2; Table S13). The signals were concentrated in early interferon, inflammatory, APC-associated, and MHC-I-machinery programs. Modules representing downstream lymphocyte function, persistence, trafficking, and tumor-associated suppressive barriers were examined but were much less often detected in sampled blood.

The principal contrasts were also stable to two analytic alternatives. Across 2208 repeated-measures contrasts in four densely sampled human datasets, generalized-estimating-equation effects correlated with the primary participant-level change effects at r = 0.998, and 97.8% had the same direction; 154 were significant under both analyses, one only under the primary analysis, and 51 only under the repeated-measures model (Table S11). In GSE116975, the alternative mean z-score produced effects correlated with the rank score at r = 0.87, with 80.7% directional agreement across all 644 contrasts; 61 results were significant by both scores, 7 only by the rank score, and 13 only by the z-score (Table S10). These sensitivities support the central early-response comparisons but do not make every individual module result interchangeable across methods.

### 3.2. AS03-Associated Early Responses Reproduce Across Trials; AS01- and MF59-Associated Effects Are Large in Their Specified Comparisons

GSE116975 directly compared five randomized arms containing identical hepatitis B surface antigen. Twenty-four hours after dose 2, AS01B, AS01E, and AS03 increased the type I interferon-associated score relative to aluminum salt by 9.6 (95% CI, 7.2–11.9), 8.6 (6.8–10.4), and 7.5 (5.7–9.3) percentile points, respectively (Table 2). The corresponding AS04 effect was 0.03 (−1.30 to 1.37). Interferon-γ-associated transcription, complement, inflammasome/IL-1, and pyroptosis/DAMP modules changed in the same early window, supporting a coordinated systemic response rather than reliance on a single module.

Two additional randomized H5N1 trials reproduced the AS03-associated response. In GSE102012, AS03 increased the interferon-associated score by 9.4 percentile points in whole blood 24 h after dose 2. In GSE112293, the corresponding estimates were 5.4 points in whole blood and 6.2 in PBMCs, with the compartments analyzed separately. MF59 increased the same score by 3.2 (1.2–5.1) points relative to antigen-matched unadjuvanted influenza vaccine in GSE124533. The antigen-matched designs hold antigen and injection schedule constant and, therefore, support a formulation-associated increment; they do not resolve the molecular source of that increment.

The AS04-versus-aluminum comparisons showed little detectable separation in sampled blood. Across 161 comparisons, estimates ranged from −1.6 to +1.6 percentile points, including the early innate modules; the type I interferon-associated estimate at 24 h after dose 2 was 0.03 (−1.30 to 1.37). In GSE124533, an aluminum-containing hepatitis B vaccine also showed little detectable change relative to the placebo in the systemic transcriptomic readout. That comparison does not isolate aluminum because antigen and formulation both differ. These results concern systemic blood transcription under the sampled conditions and do not establish the magnitude of local injection-site responses.

### 3.3. Yellow Fever 17D Produced a Prolonged, Multicomponent Response, but Duration Was Not Isolated as the Cause

**Figure 3:**
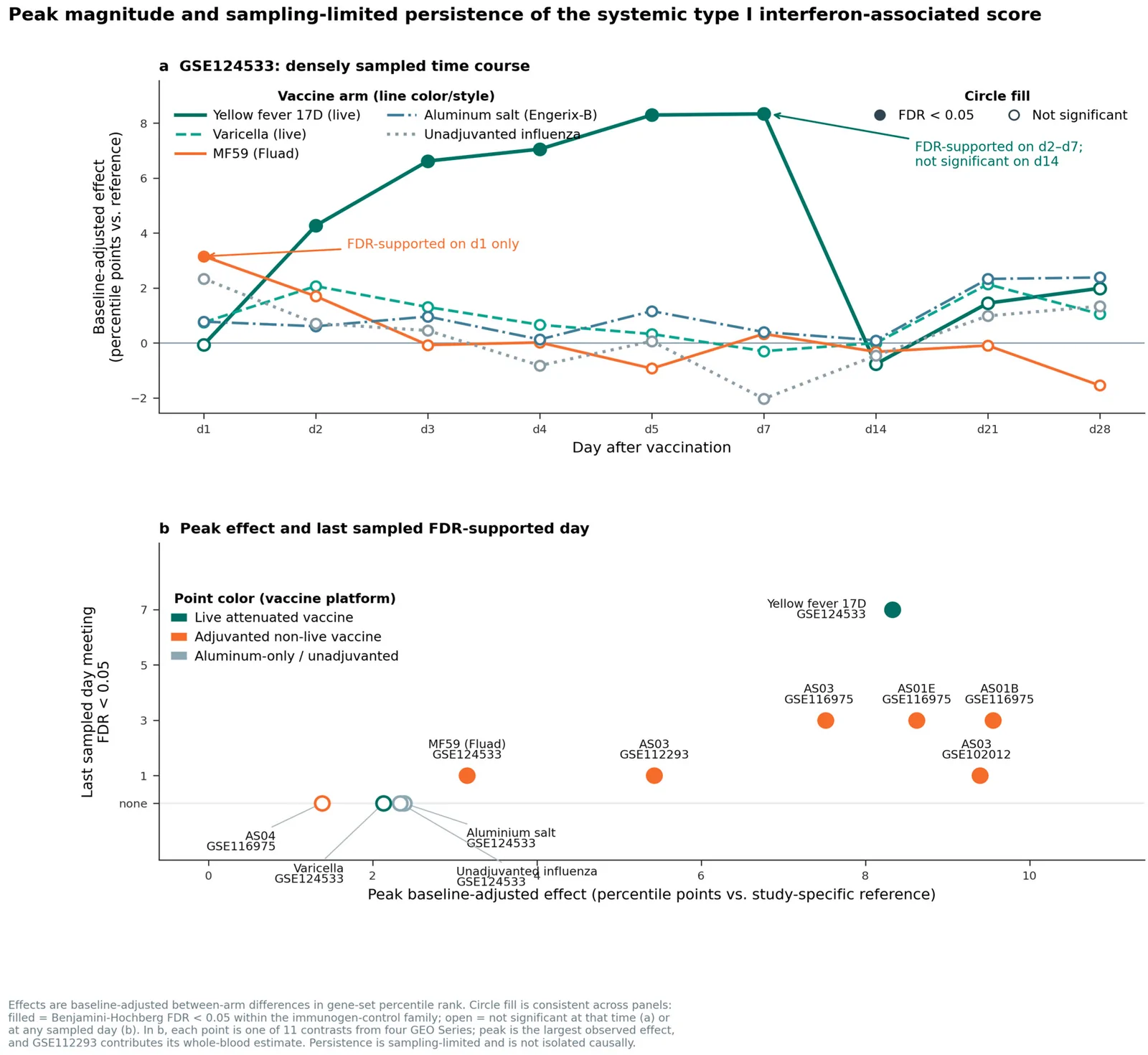
**Peak magnitude and sampling-limited persistence of the systemic type I interferon-associated score.** (**a**) Dense longitudinal sampling in GSE124533, with each point showing the baseline-adjusted between-arm difference at that time. MF59-adjuvanted influenza is compared with antigen-matched unadjuvanted influenza; yellow fever 17D, varicella, Engerix-B, and unadjuvanted influenza use the protocol-defined pooled placebo reference. Line color and style identify each formulation-control comparison, and every circle’s edge color matches its line. Filled circles meet the within-immunogen Benjamini-Hochberg FDR threshold (q < 0.05); open circles do not. (**b**) For 11 formulation-control contrasts across four GEO Series, the horizontal coordinate is the largest observed between-arm difference and the vertical coordinate is the last sampled day meeting the same FDR threshold; open circles at ‘none’ indicate that no sampled day met the threshold. Point color denotes vaccine platform: green, live attenuated; orange, adjuvanted non-live; and gray, aluminum-only or unadjuvanted. Labels identify the GEO accession, including the three AS03 comparisons. GSE112293 included separate whole-blood and PBMC analyses; the whole-blood value is shown. Persistence is descriptive and bounded by each dataset’s sampling schedule. Yellow fever 17D differs from nonreplicating formulations in replication, antigen persistence, innate inputs, and tissue distribution; the figure does not isolate response duration as a causal mechanism.

Daily sampling in GSE124533 permitted a direct descriptive comparison of the kinetics on the same platform. Yellow fever 17D increased the type I interferon-associated score from days 2–7, reaching 8.3 (7.0–9.7) percentile points on day 7; by day 14, the between-arm difference was no longer significant under the available sample size and schedule. MF59 reached 3.2 points on day 1; by day 3, the between-arm difference was no longer significant. AS01- and AS03-containing formulations in other studies reached similar or larger peaks but did not have an FDR-supported systemic difference at day 7. Figure 3 summarizes these observed sampling windows.

The difference cannot be attributed to duration alone. Yellow fever 17D is replication competent and differs simultaneously in viral antigen production, antigen persistence, innate ligands, cell tropism, and tissue distribution. Varicella, also a live vaccine, did not show the same systemic trajectory in this dataset, indicating that replication competence alone was insufficient to reproduce the yellow fever 17D pattern under these study conditions. Yellow fever 17D, therefore, produced a sustained, multicomponent trajectory, but the analysis cannot identify which correlated feature caused the later interferon-γ-associated, APC-costimulation/cDC1-associated, trafficking-associated, or GC/plasmablast-associated transcriptional changes.

### 3.4. APC-Associated Transcription Increases, but Functional Cross-Presentation Remains Unmeasured

AS01B, AS01E, AS03, and MF59 increased two modules relevant to antigen presentation in their responsive comparisons. At 24 h after dose 2 in GSE116975, the APC-costimulation/cDC1-associated score rose by 4.0, 4.1, and 3.3 percentile points with AS01B, AS01E, and AS03 relative to aluminum; the AS01B estimate was 4.04 (2.31–5.78). MHC-I antigen-processing and presentation machinery rose by 1.5–2.3 points; the AS01B estimate was 2.17 (1.59–2.75). AS03 and MF59 produced concordant changes in independent trials, whereas AS04 remained near the aluminum reference.

Participant-level associations linked these modules to the interferon-associated response. Unadjusted correlations were 0.81–0.82 for the APC-costimulation/cDC1-associated module and 0.87–0.92 for MHC-I machinery in the two studies with sufficient lineage data. After adjustment for a T-cell lineage index, the correlations were 0.49–0.75 and 0.66–0.82 (Table S7). Thus, the associations were attenuated but persisted after adjustment for the T-cell lineage index. This analysis does not identify the expressing cell, prove migration or antigen uptake, demonstrate peptide–MHC display, or establish functional cross-presentation.

### 3.5. Later Lymphocyte-Associated Programs Were Largely Undetected in Blood

The live-vaccine comparison showed the clearest later transcriptional sequence. Yellow fever 17D was followed by interferon-γ-associated transcription on days 3–4, APC-costimulation/cDC1-associated transcription on days 3–5, trafficking-associated transcription on day 7, and a GC/plasmablast-associated signal on day 14. A smaller GC/plasmablast-associated signal was present at day 7 in two AS03 trials (1.6 percentile points in each). Beyond these observations, the adjuvant studies did not show a reproducible sustained positive blood signal across the Th1/IL-12, CD8-effector/cytotoxic, NK/IL-15, naive/memory/IL-7, or trafficking modules at later sampled times.

The absence of a reproducible later blood signal has a specific scope. It establishes that later programs were not reproducibly detected in the sampled blood transcriptomes at the sampled times. It does not establish that lymphocyte priming, germinal-center activity, or trafficking failed in draining lymph nodes, and it cannot establish whether tumor-specific cells entered a tumor because neither cancer participants nor tumor specimens were present.

### 3.6. Acute Decreases in Blood Lymphocyte Scores Are Consistent with Changes in Estimated Lineage Abundance

**Figure 4:**
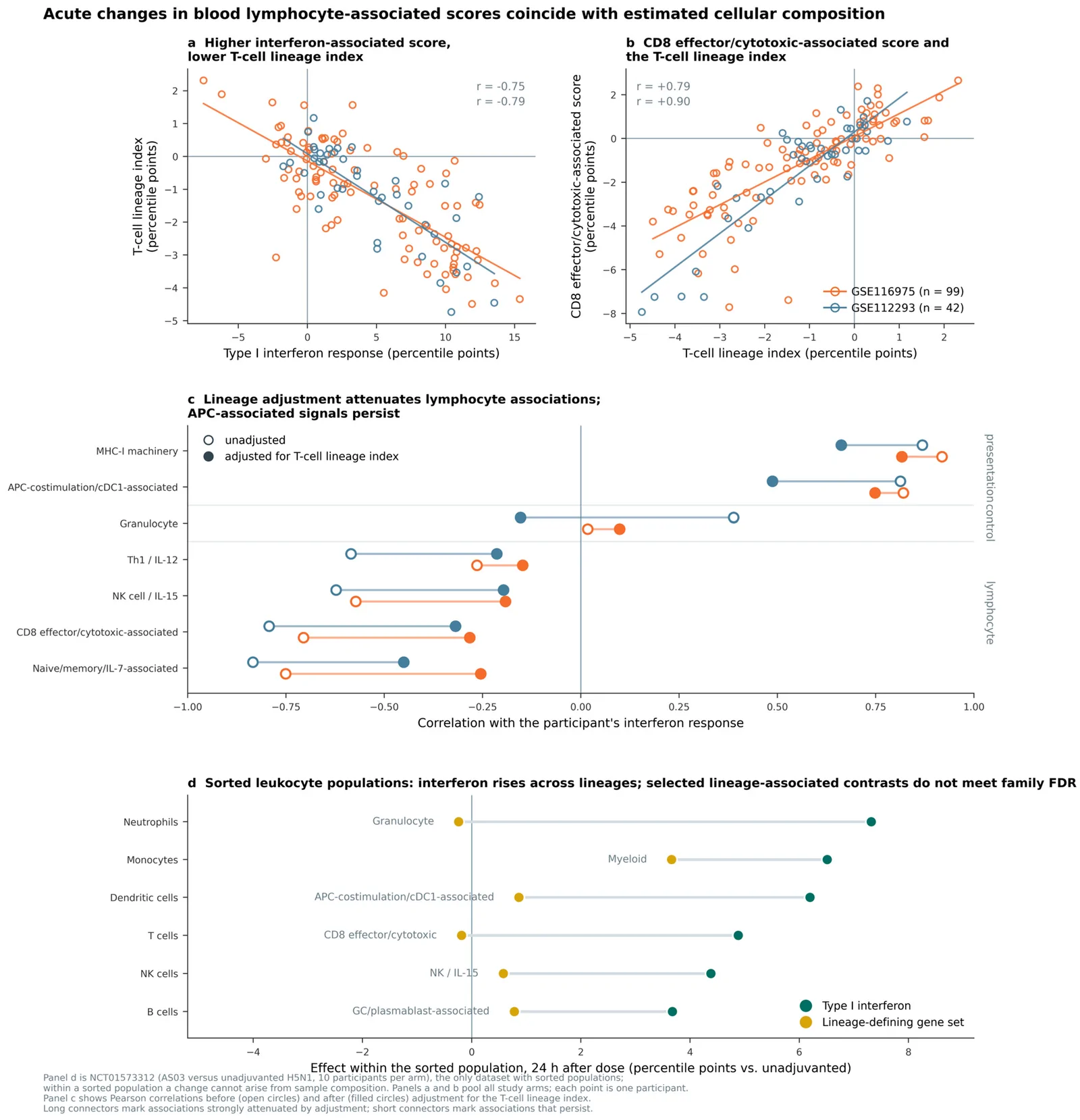
**Acute changes in blood lymphocyte-associated scores are consistent with changes in estimated lineage abundance.** Participant-level dose-2 changes relate the interferon-associated score, lineage indices, and lymphocyte-associated modules. Partial correlations show the associations after adjustment for estimated lineage abundance. The sorted-cell panel tests transcription within purified populations. The figure supports a composition-sensitive interpretation; it does not specify the destination or fate of cells that are underrepresented in blood. Orange lines and circles denote GSE116975 and blue lines and circles denote GSE112293 in panels (**a**–**c**); in panel (**c**), open circles are unadjusted and filled circles are T-cell-lineage-adjusted correlations. In panel (**d**), teal and gold circles denote type I interferon and lineage-defining gene sets, respectively.

Several lymphocyte-associated scores decreased during the early interferon response. Larger interferon-associated changes coincided with lower T-cell lineage signal (r = −0.75 and −0.79), and the CD8-effector/cytotoxic module was positively correlated with the T-cell lineage index (r = 0.79 and 0.90). Adjusting for lineage signal attenuated the association with interferon by 60% for the CD8-effector/cytotoxic module, by 67–68% for NK/IL-15, and by 46–66% for naive/memory/IL-7 (Figure 4; Table S7).

The sorted-cell trial provided a stronger test of lineage-intrinsic transcription. At day 1 after AS03-adjuvanted H5N1 vaccination, the interferon-associated score increased by 3.7–7.3 percentile points across all six sorted populations; five contrasts met the family-level FDR threshold (q = 0.007–0.039), and the NK-cell contrast was just above it (effect, +4.38; q = 0.055). In the same samples, none of the CD8-effector/cytotoxic or naive/memory/IL-7 contrasts within sorted T cells, nor the NK/IL-15 contrasts within sorted NK cells, met family-level FDR control at any sampled day. The inverse whole-blood associations with the interferon response were attenuated after lineage adjustment, and the decreases were not reproduced within the corresponding sorted populations, consistent with altered representation of circulating cell populations. The data do not distinguish margination, transient lymphopenia, sequestration, or tissue trafficking.

### 3.7. Compartment and Serum Measurements Define Two Additional Limits

The controlled mouse experiment illustrates why the absence of a later signal in blood cannot be generalized to lymphoid tissue. At 24 h, lymphocyte-associated decreases were confined to blood, whereas the draining lymph node showed a GC/plasmablast-associated increase at 72 h. Because animals were sampled terminally, these were unpaired group comparisons, and because vaccination was subcutaneous, the result is descriptive rather than a human validation. The experiment, therefore, shows that blood and draining lymph node can yield different transcriptional findings after the same immunization.

**Figure 5:**
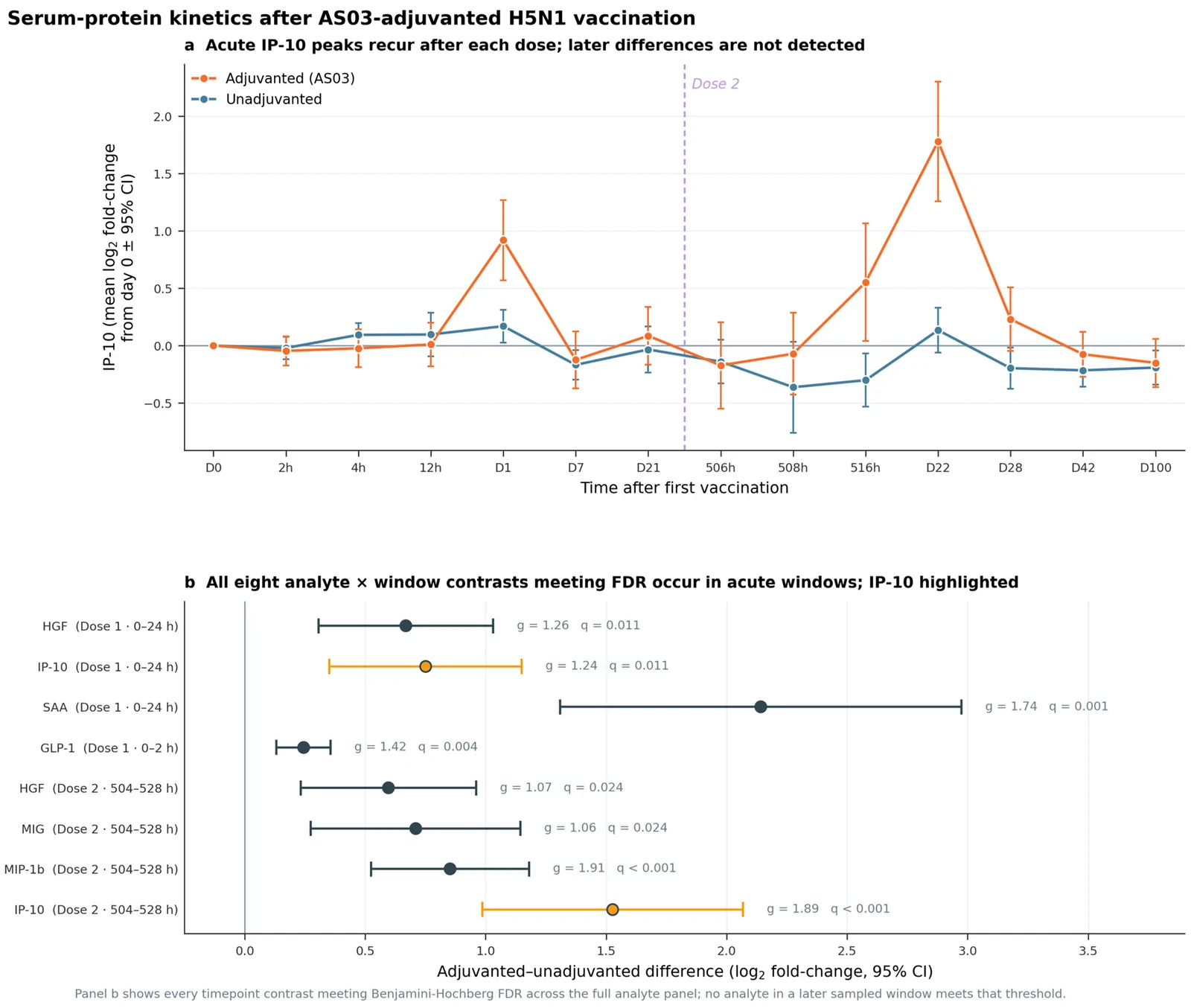
**Serum protein measurements show similarly acute systemic kinetics after AS03-adjuvanted H5N1 vaccination:** (**a**) IP-10 change by arm across 14 time points, where whiskers are 95% confidence intervals of the mean; (**b**) analyte-by-window contrasts meeting false-discovery control, shown with 95% confidence intervals. The second dose produces a larger acute pulse; persistent elevation was not detected under the sampled schedule. In panel (**a**), orange denotes the AS03-adjuvanted arm and blue denotes the unadjuvanted arm. In panel (**b**), orange denotes IP-10 and dark gray denotes the other FDR-supported analytes.

Serum IP-10 measurements confirmed that the circulating response after AS03-adjuvanted H5N1 vaccination was acute rather than sustained. In SDY1252, IP-10 increased after both doses, with a larger post-boost peak; by day 28, the mean change was near baseline (Figure 5). All eight analyte-by-window contrasts that met false-discovery control occurred in early innate windows; no later analyte met that criterion. Boosting, therefore, increased the magnitude of a second systemic pulse in this dataset, whereas persistent circulating protein activity was not detected under the available schedule.

### 3.8. One Small Cancer-Vaccine Blood Study Met the Controlled-Comparison Criteria for Poly-ICLC Reanalysis

**Figure 6:**
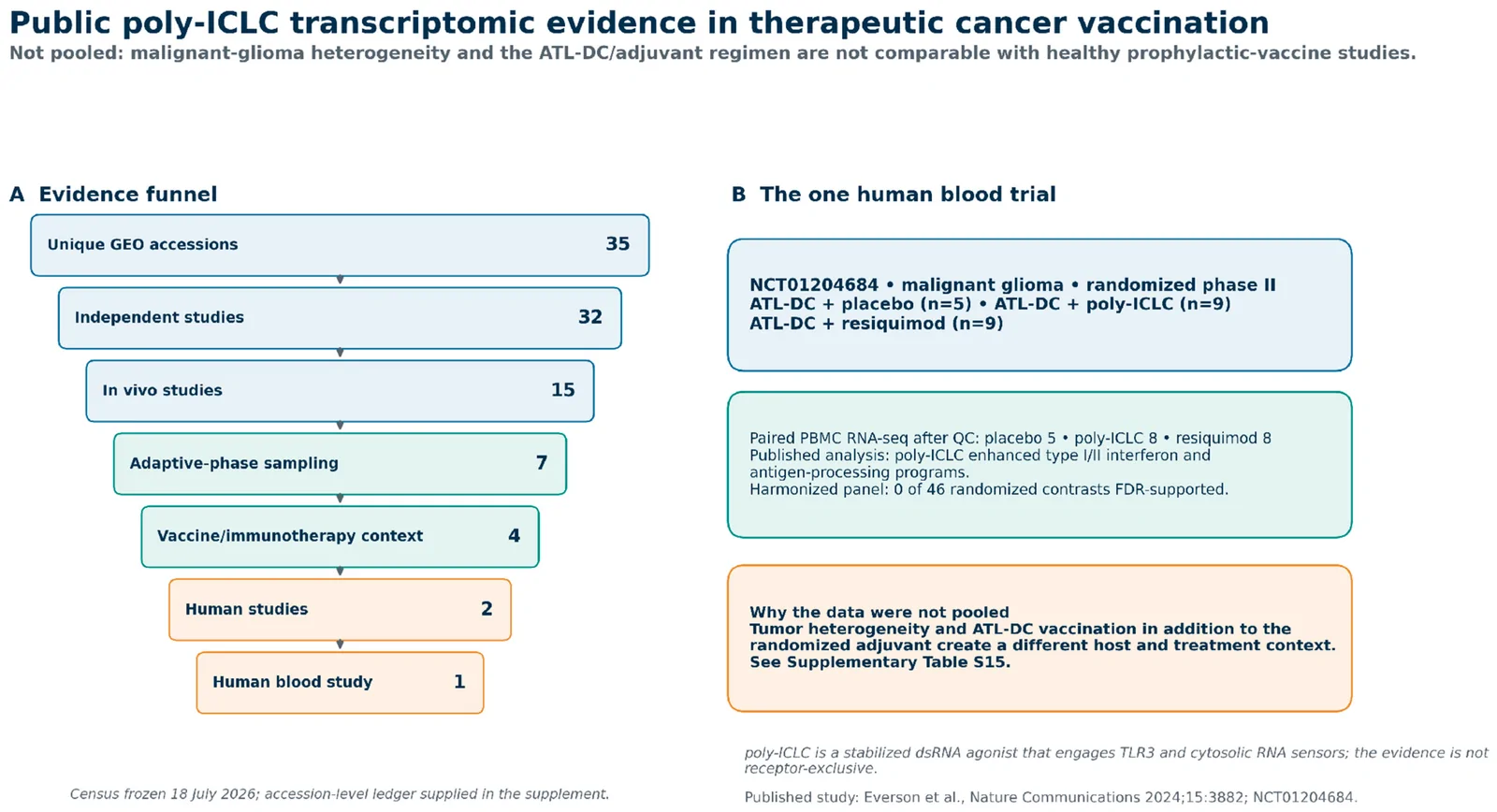
**Public poly-ICLC transcriptomic evidence in therapeutic cancer vaccination:** (**A**) availability funnel from 35 unique GEO accessions (38 eligible accession–compartment coordinates) to one human blood study meeting the controlled-comparison criterion; (**B**) NCT01204684 randomized placebo, poly-ICLC, or resiquimod on a common ATL-DC background. The published analysis identified type I/II interferon and antigen-processing programs, whereas none of the 46 randomized contrasts in the separate harmonized 23-module analysis met false-discovery control [21]. The trial was not pooled with the primary datasets because its malignant-glioma population, tumor heterogeneity, ATL-DC/adjuvant regimen, and sampling schedule were not comparable with the healthy prophylactic-vaccine studies. The accession-level census is reported in Table S14; complete module estimates are reported in Table S15.

A separate availability census was performed for public transcriptomic studies involving poly(I:C), poly-ICLC, or related double-stranded-RNA agonists because poly-ICLC is widely used in therapeutic cancer-vaccine research. The census identified 35 unique GEO accessions, corresponding to 38 eligible accession–compartment combinations and 32 independent studies. Fifteen studies were in vivo, seven sampled an adaptive-phase window, four involved vaccination or immunotherapy, two involved human participants, and one met the defined human-blood comparison criterion (Figure 6; Table S14).

The study meeting this criterion was NCT01204684, a randomized phase II trial in 23 participants with malignant glioma [21]. All participants received autologous tumor-lysate–pulsed dendritic-cell vaccination; the randomized add-on treatment was placebo (n = 5), poly-ICLC (n = 9), or resiquimod (n = 9). In the published paired bulk-PBMC analysis after quality control, the placebo, poly-ICLC, and resiquimod groups contributed 5, 8, and 8 pre/post pairs. The investigators reported increases in type I and type II interferon downstream programs and antigen-processing genes in the TLR-agonist arms [21]. Poly-ICLC is a stabilized double-stranded-RNA agonist that engages TLR3 and cytosolic RNA sensors; these data do not support receptor-exclusive attribution.

The separate harmonized reanalysis asked whether the same 23 module scores differed between the randomized add-on-treatment arms. Within the poly-ICLC arm, the prespecified 13-gene MHC-I antigen-processing and presentation machinery score increased by 4.35 percentile points from baseline (95% CI, 1.85–6.85; within-arm q = 0.011). That within-arm estimate contains the effects of ATL-DC, poly-ICLC, elapsed time, and their interactions. The randomized incremental effect of adding poly-ICLC to ATL-DC, compared with ATL-DC plus placebo, was 3.07 points (−0.62 to 6.77; Hedges g = 0.98; q = 0.247). The corresponding incremental effect of adding resiquimod was 2.73 points (−0.95 to 6.41; g = 0.87; q = 0.913).

Type I interferon-associated effects were also positive but imprecise: 2.83 points for adding poly-ICLC (−3.62 to 9.27; g = 0.62; q = 0.430) and 5.83 points for adding resiquimod (−0.73 to 12.38; g = 1.17; q = 0.913), each compared with ATL-DC plus placebo. Neither agonist-versus-placebo analysis yielded an FDR-supported module effect; none of the 46 primary module contrasts met its comparison-specific threshold. The conclusion was unchanged in the exact label-permutation, alternative mean-z-score, and timing-restricted analyses. Across the primary contrasts, the rank-score and mean-z effects were correlated (r = 0.80), with 82.6% directional agreement. The complete estimates, participant timing, quality-control exclusions, within-arm changes, and sensitivities are reported in Table S15.

The trial, therefore, provides a randomized test of adding poly-ICLC or resiquimod to an ATL-DC cancer-vaccine regimen, but it was analyzed separately from the 17 primary formulation families. Its positive interferon and MHC-I point estimates are compatible with the published analysis, but the harmonized analysis did not identify an incremental agonist effect after correction across the 23 modules. All arms received ATL-DC, so the dataset cannot estimate the effect of dendritic-cell vaccination versus no vaccine. Its small, clinically heterogeneous population and nonuniform post-treatment timing further preclude pooling with the healthy prophylactic-vaccine comparisons.

## 4. Discussion

### 4.1. Controlled Healthy-Vaccine Comparisons Establish Early Systemic Formulation Differences, Not Cancer-Vaccine Efficacy

The AS03-associated early systemic transcriptional responses were reproduced across independent healthy-vaccinee trials; AS01- and MF59-associated effects were large in their specified comparisons. AS04-versus-aluminum and aluminum-containing hepatitis B vaccine-versus-placebo comparisons showed little detectable change in sampled blood transcriptomes. The latter comparison does not isolate aluminum because antigen and formulation both differ. Antigen-matched randomized comparisons support attribution to formulation at the trial level because antigen and schedule are held constant; they do not establish which receptor pathway, cell type, or local tissue process produced the increment.

Healthy prophylactic-vaccine studies are useful because their randomized treatment and reference arms reduce several sources of variation, but that design also defines their translational limit. Participants did not have cancer, treatment-associated immune dysfunction, tumor antigen, suppressive tumor stroma, tumor-presentation defects, or antitumor endpoints. The data, therefore, estimate systemic formulation-associated differences under healthy conditions. They cannot explain why an individual cancer vaccine failed or predict clinical tumor control.

### 4.2. Yellow Fever 17D Suggests That Response Duration, Antigen Persistence, and Innate Stimulation Should Be Studied Together

Yellow fever 17D differed from formulated adjuvants in the persistence and sequence of its systemic transcriptional response. That observation is biologically informative because peak magnitude alone did not separate the groups: AS01B reached a larger early peak than yellow fever 17D, whereas yellow fever maintained a response through day 7 and showed a later GC/plasmablast-associated signal. The comparison supports reporting peak magnitude and observed persistence as separate quantities. More broadly, pan-vaccine analyses have identified recurrent early innate-response patterns while showing that their relation to later antibody responses varies with context [22].

The analysis does not show that response persistence caused the later transcriptional changes. Replication, continued antigen expression, viral innate ligands, cell tropism, and tissue distribution vary together in yellow fever 17D, and the present study cannot separate them. A testable hypothesis is that later responses depend on the timing and combination of antigen availability, innate activation, APC function, and lymphocyte priming. Matched-platform studies should vary one or more of these features directly.

The same question applies to viral platforms used for cancer vaccination. A viral vector not only delivers tumor antigen but also provides innate stimulation while the antigen is expressed. The present datasets contain no therapeutic viral-vector study, so this is a prospective hypothesis rather than a result. Direct comparison should measure viral persistence, antigen expression, local innate sensing, APC function, and antigen-specific tumor-directed immunity rather than assuming that any one feature is decisive.

### 4.3. Blood Captures Early Systemic Transcription but Not Downstream Tissue Function

The data show why the assay and compartment must be stated with every inference. Whole blood captured formulation-associated interferon, complement, and APC-associated transcription in the responsive comparisons. It did not identify the cells expressing those genes, and composite APC/MHC-I scores did not establish peptide–MHC display or productive cross-presentation. Those functions require cell-resolved phenotyping and antigen-specific assays.

The many later-time-point module–formulation cells in Figure 2 without an FDR-supported change require the same caution. Failure to detect sustained lymphocyte-associated transcription in blood is not evidence that downstream activity was absent from a draining node or tumor. It is, nevertheless, informative: if early blood activation is present while later blood programs are undetected, the next study should sample the compartments in which priming and effector function are expected. These negative blood findings, therefore, support sampling draining lymph node or tumor in follow-up studies.

Cellular composition is a second limitation that should be treated as part of the biology rather than as a statistical nuisance. Inverse associations between cytotoxic or naive/memory module changes and the interferon-associated response were attenuated after adjustment for estimated lineage scores, and the decreases were not reproduced within sorted lymphocytes, consistent with changes in circulating-cell representation. Future studies should pair bulk transcription with absolute cell counts, flow cytometry, or single-cell/sorted-cell measurements so that redistribution is not mistaken for loss of function.

GSE188620 used cell-resolved profiling that preserved cell identity and T-cell clonality. Paired single-cell RNA and T-cell receptor profiling of four selected immunological responders after tumor-lysate plus poly-ICLC vaccination identified expanded CD8+ clonotypes with effector phenotypes in blood, some of which were also detected in tumor [23]. Because this subset lacked transcriptomic control-arm samples, it cannot isolate an effect of poly-ICLC. Instead, it shows that blood can reveal downstream events when cell identity and clonality are retained, whereas aggregate PBMC transcription may obscure rare antigen-responsive populations.

### 4.4. Implications for Therapeutic Cancer-Vaccine Hypotheses

Therapeutic cancer vaccination adds requirements that are absent from these prophylactic trials. Antigen-specific cells must recognize naturally processed tumor antigen, enter tumor despite vascular and stromal barriers, retain function despite suppressive myeloid and regulatory programs, kill target cells, and persist [1,2,3,4]. None of those events can be inferred from an early blood interferon response or from an ELISpot alone.

These results identify measurements needed to test a cancer-vaccine model; they do not validate one. Several clinical formulations produced large early blood transcriptional responses under healthy conditions. The analyzed blood data did not test antigen-specific priming, tumor entry, tumor-cell killing, or persistence. Figure 2 shows that genes associated with those steps were examined explicitly, including cytotoxic effectors (PRF1, GZMB), persistence (IL7R, TCF7), trafficking (CXCR3, CXCR6), suppressive myeloid states (ARG1, TREM2), stromal exclusion (FAP, TGFB1), and abnormal vasculature (VEGFA, ANGPT2). Their limited detection in blood identifies missing evidence; it does not establish biological absence in lymph node or tumor.

NCT01204684 addresses a different cancer-specific question because every randomized arm received ATL-DC. The published study detected interferon and antigen-processing programs after ATL-DC plus TLR agonist treatment [21]. In the harmonized reanalysis, the type I interferon and MHC-I machinery point estimates were positive for adding either agonist to ATL-DC, each compared with ATL-DC plus placebo, but none met false-discovery control. The MHC-I module increased within the poly-ICLC arm, yet that change cannot be assigned to poly-ICLC because every participant also received ATL-DC and only the randomized between-arm contrast estimates the incremental agonist effect. This distinction prevents a positive pre/post change from being treated as evidence that either the dendritic-cell vaccine or the agonist was sufficient.

The trial does not answer whether ATL-DC outperformed no vaccine, whether the blood programs represented antigen-specific priming, or whether they caused tumor control. Two of five placebo post-treatment samples were also collected two weeks earlier than the active-arm samples, and restricting the reference to the three later placebo pairs reduced precision without producing an FDR-supported module effect. The interferon and antigen-processing point estimates were positive but imprecise, and PBMC transcription did not establish antigen-specific priming, tumor entry, or tumor-cell killing. Experimental evidence that combined TLR and CD40 stimulation can promote CD8 expansion remains mechanistic context [24], not proof that functional APC licensing or cross-presentation occurred in this trial.

RNA and viral platforms may change this sequence because they couple antigen delivery or expression with innate stimulation. RNA lipoplexes can target dendritic cells while engaging antiviral sensing, and ionizable lipid nanoparticles can contribute adjuvant activity and promote T-follicular-helper and germinal-center responses in preclinical systems [25,26]. The present results do not compare these platforms. They indicate which kinetics and downstream functions should be measured when such comparisons are performed.

### 4.5. Study-Derived Recommendations for Trial Monitoring

The analytic findings translate into specific measurement decisions rather than a generic call for “more immune monitoring” (Table 3). First, peak magnitude and observed persistence should be reported separately because similar peaks can have different trajectories. Second, early blood should be paired with a later draining-node or tumor measurement because later programs may be compartment restricted. Third, transcriptional associations should be corroborated at the relevant functional level. Fourth, sampling should continue across the full booster series, because the serum data show that a larger second pulse is not equivalent to a longer pulse.

### 4.6. Limitations

The primary limitation is biological scope. The 17 primary formulation questions came from prophylactic vaccination in healthy participants and contained no tumor antigen, cancer treatment, tumor biopsy, direct tumor-cell killing, response, or survival. The separate poly-ICLC trial involved 23 participants with malignant glioma and supplied a randomized cancer-vaccine PBMC comparison, but its sample size, disease heterogeneity, common ATL-DC background, absence of a no-vaccine arm, and nonuniform post-treatment timing preclude quantitative pooling with the healthy trials and limit attribution within that study. The mouse draining-node observation demonstrates compartment specificity but does not substitute for paired human blood, lymph-node, and tumor samples.

The second limitation is measurement. Bulk blood transcription combines cell abundance and cell state; the sorted-cell validation included only one adjuvant and 20 participants. Composite modules are not cell-specific, and the APC, GC/plasmablast, myeloid, stromal, and vascular scores do not prove the corresponding cellular process. Sampling schedules bound every persistence estimate, and most studies evaluated one or two doses rather than a prolonged therapeutic series.

The third limitation is analytic selection. The study was a targeted comparative reanalysis, not a preregistered systematic review or meta-analysis. Influenza studies predominate because they supplied the required antigen-matched randomized arms and dense early sampling, not because influenza is biologically privileged for oncology. The evidence cutoff was 15 July 2026, and the query was rerun on 23 August 2026 to reproduce the record census. GSE286042 was first released after the cutoff, was logged but not analyzed, and requires a distinct normalization and hierarchical analysis plan for prospective integration.

The fourth limitation concerns causal interpretation. The yellow fever comparison cannot separate response persistence from viral replication, antigen persistence, innate-ligand diversity, or tissue distribution. Similarly, antigen-matched formulation contrasts estimate the incremental effect of formulation but do not establish a specific pattern-recognition mechanism. The analysis, therefore, supports conclusions about observed blood kinetics and transcriptional associations, not receptor-level mechanisms.

## 5. Conclusions

The AS03-associated early systemic transcriptional responses were reproduced across independent healthy-vaccinee trials, and AS01- and MF59-associated effects were large in their specified comparisons. These formulations also increased APC-costimulation/cDC1-associated transcription and MHC-I antigen-processing machinery in responsive comparisons. Yellow fever 17D produced a prolonged, multicomponent response, although this analysis cannot identify response duration as the cause of its later transcriptional changes.

In the separate poly-ICLC cancer-vaccine trial, the incremental interferon and MHC-I point estimates for adding either TLR agonist to ATL-DC were positive, but no primary module contrast met false-discovery control. The trial, therefore, estimates randomized incremental agonist effects; it does not establish an agonist effect, ATL-DC efficacy versus no vaccine, or a causal relationship between a blood program and tumor control.

For therapeutic cancer-vaccine research, the central result is the distinction between what early blood can and cannot measure. Early blood can establish an early systemic transcriptional response; it cannot establish lymph-node priming, tumor access, naturally processed antigen recognition, target-cell killing, or memory. Prospective trials should, therefore, pair early systemic kinetics with later tissue and antigen-specific functional measurements and should evaluate how viral, RNA, and sustained-expression platforms combine antigen expression, innate stimulation, and response duration rather than treating them only as antigen-delivery vehicles.

## Figures and Tables

**Table 1:** Public transcriptomic datasets included in the submitted cohort set.

Study	Design and Comparison	Samples/Participants	Sampling	Compartment/Platform
GSE116975 (NCT00805389) [6]	Randomized hepatitis B surface antigen with AS01B, AS01E, AS03, AS04, or aluminum salt; aluminum reference	924 arrays; 112 participants	3–6 h, 24 h, and later windows after two doses	Whole blood; Affymetrix U133 Plus 2.0
GSE112293 [12]	Randomized AS03-adjuvanted versus unadjuvanted H5N1	898 scored profiles; 893 assigned to the analyzed whole-blood/PBMC strata; 42 participants	2, 4, 12, and 24 h; days 7–100 after two doses	Whole blood and PBMCs analyzed separately; Affymetrix Human Gene 2.1 ST
GSE102012 [13]	Randomized AS03-adjuvanted versus unadjuvanted H5N1	384 arrays; 49 participants	Days 1, 3, and 7 after two doses	Whole blood; Affymetrix HT HG-U133+ PM
GSE124533 (CRC305A–C) [8]	Randomized vaccines and protocol-matched placebo; MF59 influenza compared with antigen-matched unadjuvanted influenza	1606 arrays; 122 participants	Daily on days 1–5, then days 7, 14, 21, and 28	Whole blood; Agilent 8 × 60 K
GSE74975 [7]	Randomized MF59-adjuvanted versus unadjuvanted influenza in children	230 arrays; 82 participants	Cohort-staggered days 1, 3, or 7 after boost; day 56	Whole blood; Affymetrix HT HG-U133+ PM
PMID 28099485 (NCT01573312) [14]	Randomized AS03-adjuvanted versus unadjuvanted H5N1	825 profiles; 20 participants	Days 0, 1, 3, 7, and 28	Six sorted leukocyte populations; RNA sequencing
GSE85339 [15]	Controlled H56 antigen alone or with GLA-SE, aluminum salt, CAF01, or IC31	293 arrays; 3–4 mice/group/time	6, 24, 72, and 168 h	Whole blood and draining node; Agilent mouse 8 × 60 K

**Table 2:** **Major baseline-adjusted effects with 95% confidence intervals.** Effects are between-arm differences in participant-level change, in percentile-rank points. Full results and adjusted probabilities appear in Tables S5 and S11.

Study/Contrast	Module and Time	Effect (95% CI)	Hedges g
GSE116975, AS01B vs. aluminum	Type I IFN-associated, 24 h after dose 2	9.55 (7.17–11.94)	3.02
GSE116975, AS01E vs. aluminum	Type I IFN-associated, 24 h after dose 2	8.62 (6.85–10.40)	3.21
GSE116975, AS03 vs. aluminum	Type I IFN-associated, 24 h after dose 2	7.52 (5.71–9.32)	2.25
GSE116975, AS04 vs. aluminum	Type I IFN-associated, 24 h after dose 2	0.03 (−1.30–1.37)	0.02
GSE124533, MF59 vs. unadjuvanted influenza	Type I IFN-associated, day 1	3.15 (1.25–5.05)	1.02
GSE124533, yellow fever 17D vs. placebo	Type I IFN-associated, day 2	4.27 (2.72–5.82)	1.73
GSE124533, yellow fever 17D vs. placebo	Type I IFN-associated, day 7	8.33 (6.97–9.69)	3.85
GSE116975, AS01B vs. aluminum	MHC-I machinery, 24 h after dose 2	2.17 (1.59–2.75)	2.79
GSE116975, AS01B vs. aluminum	APC-costimulation/cDC1-associated, 24 h after dose 2	4.04 (2.31–5.78)	1.68

**Table 3:** Prospective therapeutic-cancer-vaccine measurements suggested by the observed results.

Observation in the Present Analysis	Defensible Inference	Measurement Required Next
AS01B, AS01E, AS03, and MF59 increase early interferon-associated transcription in their responsive comparisons	A formulation-associated systemic response occurred	Local cytokines and injection-site biology; cell-resolved source; matched vehicle/formulation studies if receptor mechanism is claimed
APC-costimulation/cDC1-associated and MHC-I-machinery scores increase	The module genes increased in blood, but cell identity and antigen-presentation function were not measured	APC phenotype and migration; antigen uptake; peptide–MHC display; functional cross-presentation
Acute cytotoxic/naive-memory scores fall in whole blood but not sorted lymphocytes	The pattern is consistent with a change in circulating-cell representation	Absolute counts, flow cytometry, sorted/single-cell transcription, and tissue trafficking measurements
Sustained later lymphocyte-associated programs are not detected in blood	The sampled systemic assay did not demonstrate the named later lymphocyte-associated transcriptional changes	Draining-node and tumor sampling; antigen-specific clonotypes, phenotype, cytokine production, and killing assays
Yellow fever 17D shows a sustained multicomponent trajectory	Duration accompanies, but is confounded with, replication, antigen persistence, innate ligands, and distribution	Matched platform experiments that manipulate these variables and measure downstream function
A booster increases the second IP-10 peak without a sustained serum signal	Peak amplitude and persistence are distinct	Sampling after every dose through late time points; link kinetics to clonal expansion, tumor entry, killing, and memory

## Data Availability

Primary transcriptomic data are available from GEO under GSE116975, GSE112293, GSE102012, GSE124533, GSE74975, and GSE85339. Gene-expression quantifications for NCT01573312 (PMID 28099485) are available with the original publication. For NCT01204684, the bulk PBMC RNA-seq data analyzed here are in GSE237562; GSE237579 is the linked single-cell subseries under parent accession GSE237581. Serum protein data are available through ImmPort SDY1252. The submitted analysis, derived scores, code, supporting documentation, and final Supplementary Materials workbook are archived at https://zenodo.org/records/22133852.

## Supplementary Materials

The following supporting information can be downloaded at: https://www.mdpi.com/article/10.3390/vaccines14090792/s1, Table S1: Dataset disposition and GSE112293 compartment-assignment audit (898 scored; 893 assigned to analyzed strata); Table S2: Definitions, complete membership, reporting class, and provenance of the 23 modules; Table S3: Cell-lineage identity markers used as composition covariates; Table S4: Pairwise GSE116975 adjuvant-arm comparisons across early innate modules and 24 h windows; Table S5: All 3725 tabulated rows (3633 evaluable), with primary and pooled-within-study multiplicity, family labels, and Hedges g; Table S6: Covariate and baseline-balance audit by study and arm; Table S7: Composition-adjustment and sorted-population analyses supporting Figure 4; Table S8: All 253 pairwise module-overlap comparisons (298 memberships; 290 unique genes); Table S9: Module-specific gene coverage on the four human array platforms; Table S10: Alternative mean z-score sensitivity for all 644 GSE116975 contrasts; Table S11: Repeated-measures generalized-estimating-equation sensitivity for 2208 contrasts; Table S12: Exact GEO query, evidence-cutoff and rerun ledger, and record-level eligibility decision for 385 records; Table S13: Complete 23-module × 17-family evidence matrix, representative genes, and cancer-vaccine rationale used in Figure 2; Table S14: Poly(I:C)/poly-ICLC search methods, availability funnel, and census of 35 unique GEO accessions used in Figure 6; Table S15: NCT01204684 paired bulk-PBMC 23-module analysis: participant timing, quality control, module coverage, randomized contrasts, exact permutation tests, alternative score, and timing sensitivity.

## Abbreviations

APC, antigen-presenting cell; AS, adjuvant system; ATL-DC, autologous tumor-lysate–pulsed dendritic cell; cDC1, type 1 conventional dendritic cell; CI, confidence interval; FDR, false discovery rate; GC, germinal center; GEE, generalized estimating equation; GEO/GSE, Gene Expression Omnibus/GEO Series; H5N1, influenza A(H5N1); IFN, interferon; IL, interleukin; IP-10, interferon-γ-induced protein 10 (CXCL10); LNP, lipid nanoparticle; MHC, major histocompatibility complex; MF59, squalene-based oil-in-water emulsion; NK, natural killer; PBMC, peripheral blood mononuclear cell; RNA, ribonucleic acid.
